# Guiding People to Interpret Their Experienced Difficulty as Importance Highlights Their Academic Possibilities and Improves Their Academic Performance

**DOI:** 10.3389/fpsyg.2018.00781

**Published:** 2018-05-25

**Authors:** Daphna Oyserman, Kristen Elmore, Sheida Novin, Oliver Fisher, George C. Smith

**Affiliations:** ^1^Department of Psychology, Dornsife Mind and Society Institute, University of Southern California, Los Angeles, CA, United States; ^2^Department of Psychology, Institute for Social Research, University of Michigan, Ann Arbor, MI, United States; ^3^Department of Psychology, Cornell University, Ithaca, NY, United States; ^4^Department of Developmental Psychology, Utrecht University, Utrecht, Netherlands

**Keywords:** self and identity, possible self, motivation, academic achievement, interpretation of experienced difficulty, metacognition, social class

## Abstract

Does experiencing difficulty bolster or undermine future self-images, strategies to get there and actual performance? We build on four insights from prior research to predict that accessible interpretation-of-experienced-difficulty mindset shapes identity and performance. First, people have two different interpretation-of-experienced-difficulty mindsets available in memory; their difficulty-as-impossibility mindset focuses attention on difficulty as implying low odds and their difficulty-as-importance mindset focuses attention on difficulty as implying high value. Second, people are sensitive to contextual cues as to which mindset to apply to understand their experienced difficulty. Third, people apply the mindset that comes to mind unless they have reason to question why it is “on-the-mind.” Fourth, social class can be thought of as a chronic context influencing how much people endorse each interpretation-of-experienced-difficulty mindset. We used subtle primes to guide participants’ attention toward either a difficulty-as-importance or a difficulty-as-impossibility mindset (*N* = 591). Participants guided toward a difficulty-as-importance mindset performed better on difficult academic tasks (Studies 1, 2) than participants guided toward a difficulty-as-impossibility mindset; whether they had more school-focused possible identities and linked strategies depended on sample (Studies 3, 4). For college students, the effect of guided interpretation-of-experienced-difficulty mindset was not moderated by how much participants agreed with that mindset (Studies 1, 3, 4). College students mostly disagreed with a difficulty-as-impossibility mindset, but making that mindset accessible undermined their performance and sometimes their possible identities anyway. In contrast, middle school students (a younger and lower social class sample) were more likely to agree with a difficulty-as-impossibility mindset. In this sample (Study 2), we found an effect of mindset endorsement: agreeing that difficulty implies importance and disagreeing that difficulty implies impossibility improved performance. This study had a control group. Control group participants not guided to use a particular interpretation-of-experienced-difficulty mindset performed no differently than participants guided toward a difficulty-as-impossibility mindset. Results suggest that people may chronically act as if they are using a difficulty-as-impossibility mindset and may benefit from being guided to consider that experienced difficulty might imply task importance. Effect of accessible mindset on salience of academic possible selves was not stable, accessible mindset mattered in one university sample but not the other.

## Introduction

“There is no use trying,” said Alice, “one can’t believe impossible things.” (Carroll, 1899, p. 90)

Like Alice, people often infer meaning from their experiences of difficulty (Koriat, 1993; Schwarz, 2015; Schwarz and Strack, 2016). Identity-based motivation theory predicts that people have available in memory two different ways to make sense of what experienced difficulty implies for who they are and what they should do (Oyserman, 2007; Oyserman et al., 2017). One way to make sense of experienced difficulty is Alice’s difficulty-as-impossibility mindset. This mindset draws attention to the odds of success, so people might think: “Might this be impossible for me?” or even “Who am I kidding, *I* will not be able to succeed at this; any sacrifice I might make is not worthwhile.” Given their concern to not waste their time on impossible things, they may be particularly sensitive to not putting in too much effort. The second way to make sense of experienced difficulty is by using a difficulty-as-importance mindset. This mindset draws attention to the value of success, so people might think: “Might this be important for me?” or even “No pain, no gain, this is important for *me*, sacrifice is worth it.” Given their interest in value, they may be particularly energized by difficulty. Sometimes people draw on Alice’s difficulty-as-impossibility mindset, then they do not invest energy in impossible things; other times people draw on a difficulty-as-importance mindset, then they become energized by the possible value of difficult things. We test two predictions: First, that the interpretation-of-experienced-difficulty mindset that is “on the mind” (accessible) influences actual performance and what people imagine is possible for themselves. Second, that this effect of being “on the mind” does not depend on how much people explicitly agree with the implications of an accessible mindset interpretation-of-experienced-difficulty.

### Motivational Mindsets

People have the capacity to hold multiple motivational mindsets in memory; for example, they have available to them both approach and avoidance motivational systems (Chen and Bargh, 1999), both promotion and prevention motivational systems (Liberman et al., 1999), and both fixed and growth motivational systems (Lou and Noels, 2016; Van Tongeren and Burnette, 2016). People do not use all the information that is available to them in memory at any particular moment in time. Instead, they use the subset of all available information that is accessible “on-the-mind” in the moment (Higgins et al., 1977; Srull and Wyer, 1979; Higgins, 1989, 1996; Schwarz, 2015; Schwarz and Strack, 2016). What is accessible is assumed to be accessible for a reason, and people use accessible information unless they have reason to set it aside as irrelevant (Bargh et al., 1996). In each case, research has documented that which motivational mindset people use is a function of which is “on-the-mind” (accessible) at the moment of judgment. Taken together, prior research clarifies that a mindset can be on the mind either because it is chronically “on-the-mind” or because it is momentarily “on-the-mind” (e.g., for promotion and prevention, Liberman et al., 1999; Wan and Wyer, 2015; Lou and Noels, 2016; for growth and fixed mindset, Van Tongeren and Burnette, 2016; for difficulty-as-importance and difficulty-as-impossibility, Smith and Oyserman, 2015; Aelenei et al., 2016).

### Two Distinct Interpretation-of-Experienced-Difficulty Mindsets Are Available in Memory

Identity-based motivation theory starts with the observation that almost anything a person does can feel easy or difficult and that while people may or may not infer something from these feelings, they often do (Oyserman et al., 2017). A large body of work demonstrates that people make inferences based on their metacognitive experiences unless given reason not to, even if relevant content is also available (for reviews, see Oppenheimer, 2008; Schwarz, 2015). Identity-based motivation theory provides a theoretical framework for how this works in the domain of identity, predicting that people are motivated to act and interpret their experiences in ways that feel congruent with their identities (Oyserman, 2007, 2009). At the same time, which identities come to mind and what these identities are taken to mean and imply for behavior and interpretation of difficulty is dynamically constructed in the moment (Oyserman, 2015). Experienced difficulty can imply low odds of success, a difficulty-as-impossibility mindset: “I don’t know this (or cannot learn it), this is not for me.” This interpretation seems common. For example, presumably because of their chronically accessible difficulty-as-impossibility mindsets, people do not use learning strategies that feel difficult even if told that these difficult strategies are more effective for learning (Kornell and Bjork, 2008; Karpicke et al., 2009; Yan et al., 2016). Experienced difficulty can also imply task value, a difficulty-as-importance mindset: “I really care about this, ‘no pain, no gain,’ this is for me.” This interpretation is less common, as shown in English-language word usage analysis (Yan and Oyserman, unpublished data). However, guiding students to imagine their academic future selves seems to help, increasing endorsement of difficulty-as-importance (Oyserman et al., 2015).

Fisher and Oyserman (2017) conducted five studies to provide descriptive information about difficulty-as-impossibility and difficulty-as-importance mindsets and how they relate to established motivational mindsets including self-efficacy, locus of control, growth mindset, grit, promotion focus, and prevention focus. They asked how much people endorsed difficulty-as-impossibility and difficulty-as-importance mindsets, whether demographics mattered, if difficulty-as-impossibility and difficulty-as-importance mindsets were correlated and whether they were distinct from other established motivational mindsets. Their results showed that on average, people agreed with difficulty-as-importance mindset (that experiencing difficulty is a signal of high value) and disagreed with difficulty-as-impossibility mindset (that experiencing difficulty is a signal of low odds). However, having low income was associated with less disagreement with difficulty-as-impossibility mindset. How much people endorsed a difficulty-as-importance mindset was not a good predictor of how much they endorsed a difficulty-as-impossibility mindset, the correlation, though negative, was small using Cohen’s (1988) rule-of-thumb. Difficulty-as-importance and difficulty-as-impossibility mindsets met criteria for discriminant and convergent validity; difficulty-as-importance and difficulty-as-impossibility mindsets are distinct from other established motivational mindsets. They are no more (and sometimes less) correlated with other established motivational mindset measures than these established measures are with one another. For example, difficulty-as-importance mindset (believing that difficulty signals value for oneself) is distinct from self-efficacy (believing that one can control their outcomes, Bandura, 1977) and growth mindset (believing that ability can be increased with effort; Dweck, 2006).

A number of studies suggest that whether a difficulty-as-impossibility or a difficulty-as-importance mindset is accessible matters for identity content, academic performance, and self-regulation. For example, participants randomized to use a difficulty-as-importance mindset were more certain about academic identities compared to participants randomized to use a difficulty-as-impossibility mindset (Smith and Oyserman, 2015; Aelenei et al., 2016). Difficulty-as-importance condition participants had better performance on academic tasks than difficulty-as-impossibility condition participants (Smith and Oyserman, 2015; Elmore et al., 2016). If they were dieters, difficulty-as-importance condition participants showed more self-control in their eating than difficulty-as-impossibility condition participants (Lewis and Earl, 2017).

### Gaps to Be Addressed

In sum, studies to date show that whether a difficulty-as-importance or a difficulty-as-impossibility mindset is “on-the-mind” matters for identity, performance, and control (Smith and Oyserman, 2015; Aelenei et al., 2016; Elmore et al., 2016; Lewis and Earl, 2017). Compared to when a difficulty-as-impossibility mindset is “on-the-mind,” when a difficulty-as-importance mindset is “on-the-mind,” students are more likely to view themselves as school-focused and perform better on school tasks. These important findings are worthy of continued study to understand whether effects are found across measures, are moderated by how much students agree with the “on-the-mind” mindset, and which pattern is more similar to what would occur without a priming task. That is our goal in the current studies. We focus on effects of accessible difficulty-as-importance and difficulty-as-impossibility mindsets on identity and school performance. We ask if effects generalize to other dependent measures and priming tasks, if effects are moderated by how much participants agree with the mindset the priming task brought to mind, and if without a priming task students seem to use a difficulty-as-impossibility mindset.

With regard to generalizing to other dependent measures, prior studies testing the effect of accessible interpretation of experienced difficulty on identity used closed-ended measures of identity (Smith and Oyserman, 2015; Aelenei et al., 2016) and a particular measure of academic performance (the Raven’s Progressive Matrices Task, Smith and Oyserman, 2015; Elmore et al., 2016). While a closed-ended academic identity measure provides a standardized assessment, it might be susceptible to social desirability effects – being asked to rate how much one agrees with a statement is different from generating that content from an open-ended probe. Replication of effects using the Raven’s is useful but generalization requires also using other standardized measures of school performance that are used in school settings.

With regard to priming tasks, the test of effects of accessible interpretation-of- experienced-difficulty mindset on difficult academic tasks relied on autobiographical recall and social comparison cues (Smith and Oyserman, 2015)^1^. What is missing is a test of whether this finding requires autobiographical memory and social comparison. Replication using a standardized priming task such as a biased scale manipulation would increase generalizability and would allow for a direct test of the possibility that effects are moderated by endorsement of accessible interpretation-of-experienced-difficulty mindset. Testing moderation is particularly important since a number of studies reveal variation in endorsement by social class (Aelenei et al., 2016; Fisher and Oyserman, 2017). Finally, prior studies document the effect of accessible difficulty-as-importance compared to difficulty-as-impossibility mindset but cannot address the question of whether when no interpretation of difficulty is primed people act as they would when a difficulty-as-impossibility mindset is accessible.

## Current Studies

We test the effect of accessible difficulty-as-importance compared to difficulty-as-impossibility mindset using a standard biased-scale manipulation (e.g., Bohner and Schwarz, 1996; Dillehay and Jernigan, 1970).^2^ This allows us to directly test if effects are moderated by endorsement of accessible mindset. In each case we predict that difficulty-as-importance condition participants will fare better than difficulty-as-impossibility condition participants and that this main effect of mindset accessibility will not be moderated by mindset endorsement.

We randomize participants into two experimental groups. One group reads and rates endorsement of a set of items describing experienced difficulty as a signal of importance (no pain, no gain). The other group reads and rates endorsement of a set of items describing experienced difficulty as a signal of impossibility. Specifically, in Studies 1 and 2 we test the effect of accessible interpretation-of-difficulty mindset on academic engagement and performance. In Study 1 we test effects of accessible interpretation-of-difficulty mindset using the Raven’s measure to replicate prior results. We predict that students in the difficulty-as-importance condition will outperform those in the difficulty-as-impossibility condition. In Study 2 we use a different difficult standardized academic task to address the question of generalizability.

In Studies 3 and 4 we test the effect of accessible interpretation-of-difficulty mindset on identity content using an ecologically valid open-ended measure of possible selves and strategies used in prior intervention studies (Oyserman et al., 2006). We predict that accessible interpretation-of-difficulty mindset has a causal impact on the number of academically focused identities and strategies to attain them students generate when asked an open-ended question about what is possible for the self. Students in the difficulty-as-importance condition will generate more school-focused possible identities and strategies to attain them than students in the difficulty-as-impossibility condition.

In two Studies (2 and 4) we randomize participants to three groups (difficulty-as-importance, difficulty-as-impossibility, no primed mindset control group). We predict that students in the difficulty-as-importance condition will outperform those in the difficulty-as-impossibility and control conditions. We explore whether or not students in the difficulty-as-impossibility condition display outcomes that differ from students in the no treatment control group.

In addition to the predicted effect of accessible interpretation-of-difficulty mindset, we explore the possibility that social class matters. We ask if prior associations of social class with endorsement of interpretation-of-difficulty mindsets translates into differences in the effectiveness of primes or to descriptive differences in likelihood of endorsing a mindset once it is made accessible.

### Samples and Analyses Plan

**Table 1** shows sample information. Sample size was limited by class size in middle school and by subject pool allocation for college samples; our goal was 50 or more participants per condition. In each study, we used regression analyses, contrast coding conditions. In Studies 1 and 3 contrast codes were (‘difficulty-as-importance’ condition as +1 and ‘difficulty-as-impossibility’ condition as -1) and we centered endorsement scores to compute condition by endorsement interaction variables. In Studies 2 and 4, we introduced a control condition and contrast coded the three conditions into two contrasts (Contrast 1: ‘difficulty-as-importance’ condition as +2/3, ‘difficulty-as-impossibility’ condition as -1/3, control condition as -1/3; Contrast 2: ‘difficulty-as-importance’ condition as 0, ‘difficulty-as-impossibility’ condition as -1/2, control condition as 1/2). The first contrast allowed us to test whether being in the ‘difficulty-as-importance’ condition changes outcomes compared to control and ‘difficulty-as-impossibility’ conditions combined; the second contrast allowed us to test whether being in the ‘difficulty-as-impossibility’ condition changes outcomes compared to being in the no prime control condition. Figures present results graphically.

**Table 1:** Sample information by study.

Study	*N*	Social Class	% Male	Age (*SD*)	% White	% Black	% Latino	% Asian	% Other
1	129	High	58%	18.85 (0.93)	68.0	6.3	3.9	12.5	3.9
2	163	Low	45%	11.66 (0.58)	3.1	67.9	4.3	0.6	22.8
3	110	High	51%	18.95 (1.14)	65.5	2.7	2.7	17.3	7.2
4	189	High	32%	19.90 (1.68)	39.7	4.8	10.1	27.0	6.9

*High = Higher resource, either a top ranked private or a public flagship university. Low = lower resource, a middle school in a lower income town near Detroit, MI, United States. Other, responded other or responded “multiple.” International students comprised 5.5% of students in Study 1, 4.5% of students in Study 3, and 11.6% of students in Study 4.*

Prior to data collection, we obtained university human subjects approval for studies 1–3 from the University of Michigan’s Institutional Review Board and for study 4 from the University of Southern California’s Institutional Review Board. For Studies 1, 3, and 4 consent forms and procedures for adult college student participants (age 18 or over) received approval from the IRB board; in Study 2, forms and procedures for both parent consent and youth participant assent were all approved by the IRB board as well as by administrators and teachers at the middle school.

Data and syntax for all studies are available on the OPEN ICPSCPSR website^3^. We did not predict or find moderating effects of demographics on condition, so we include demographics in analyses only if they are associated with the dependent variable as omitting them if they influence the dependent variable would provide a distorted picture of the effect of the priming task. In Studies 3 and 4 our dependent variable were count scores based on coding of open-ended responses. That means that the dependent variable did not have a fixed range, and that any outliers might skew results, making them less likely to be stable across studies. Therefore we looked for outliers (Standardized Pearson’s residual greater than 3.0) and omitted any such responses from analyses. For completeness analyses are presented again with outliers.

### Study 1

#### Sample

University of Michigan undergraduates (*N* = 131) participated for course credit, coming to the lab and working on a computer using Qualtrics software.

#### Procedure

Participants were randomly assigned to condition (difficulty-as-importance, difficulty-as-impossibility) which entailed reading and rating (1 = *strongly disagree*, 6 = *strongly agree*) four statements. Statements were about interpreting difficulty and identical with one exception, the word important or impossible. The word important appeared in one statement set (*n* = 66, e.g., *Some school tasks feel easy and some feel difficult. My gut tells me that if it feels difficult, it is important for me*). The word impossible appeared in the other statement set (*n* = 65, e.g., *Some school tasks feel easy and some feel difficult. My gut tells me that if it feels difficult, it is impossible for me)*. The dependent variable was the 12-item Raven’s Advanced Progressive Matrices (RAPM, Raven et al., 1996) short version (Bors and Stokes, 1998), commonly used to assess fluid intelligence. Specifically, participants were shown 12 eight-image patterns in a 3 × 3 grid with the ninth image in the bottom right corner missing. They then selected one of eight options to complete each pattern and proceeded to the next pattern, time spent on each item was recorded using the Qualtrics software. Demographics (gender, age, race) were obtained at the end of the study. Two participants, one in each condition, reported not understanding instructions and so were dropped (*n* = 129 for analysis).

We calculated efficiency (number of correct responses per minute spent on the RAPM), which allowed us to account for both accuracy and time spent on the task (*M* = 1.10, *SD* = 0.61). Female participants were significantly more efficient than males (*b* = 0.23, *p* = 0.038, 95% CI [0.01, 0.44]) so gender was included as a covariate in the regression analyses predicting efficiency.

#### Results

**FIGURE 1:**
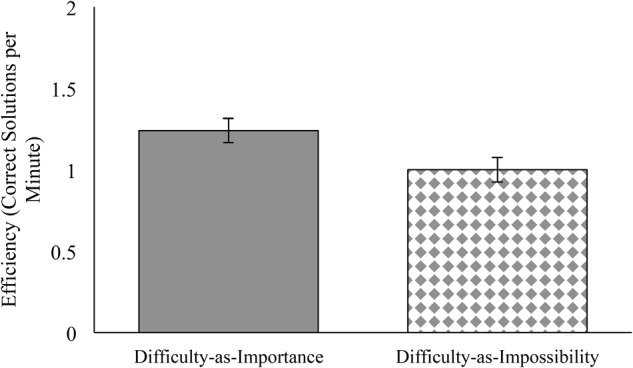
Study 1: Efficiency (correct solutions per minute) on Raven’s Advanced Progressive Matrices by Condition (Difficulty-as-Importance, Control, Difficulty-as-Impossibility). Estimated marginal means include adjustments for covariates. Error bars represent standard errors.

The prediction that difficulty-as-importance condition improves performance relative to difficulty-as-impossibility condition was supported. Students in the difficulty-as-importance condition (*M* = 1.24, *SE* = 0.07) were more efficient, solving more problems correctly per minute than students in the difficulty-as-impossibility condition (*M* = 1.00, *SE* = 0.08), *b* = 0.12, *p* = 0.023, 95% CI [0.02, 0.22], *d* = 0.40. This effect was robust, remaining when the effect of condition on efficiency was assessed without controlling for the significant individual difference by gender described in the preliminary analyses, *b* = 0.12, *p* = 0.022, 95% CI [0.02, 0.23] as depicted in **Figure 1**. The effect is also robust to analytic technic: calculating Raven’s performance as percentage correctly solved (ignoring time) also reveals that students in the difficulty-as-importance condition solved a larger percentage of the problems correctly (*M* = 52.56%, *SD* = 23.89) than students in the difficulty-as-impossibility condition (*M* = 43.36%, *SD* = 21.73; *b* = 4.60, *p* = 0.024, 95% CI [0.62, 8.58]).

As shown in **Table 2**, most participants in the difficulty-as-importance condition agreed that difficulty means importance (*M* = 3.98 *SD* = 0.99) and most participants in the difficulty-as-impossibility condition disagreed that difficulty means impossibility (*M* = 2.29, *SD* = 0.75). If endorsement mattered, we should have found that rejecting difficulty-as-impossibility and accepting difficulty-as-importance had parallel effects. To test this possibility, we entered mean endorsement scores into our regression equation, finding neither a main (*b* = 0.09, *p* = 0.123, 95% CI [-0.03, 0.21]) nor an interaction effect of endorsement, *b* = 0.04, *p* = 0.506, 95% CI [-0.08, 0.17]. The implication is that when priming tasks are subtle, people are sensitive to the implications of the interpretation-of-difficulty mindset that is accessible and use it in considering what is possible for them even if they do not explicitly agree with this mindset.

**Table 2:** Percentage endorsement of guided interpretation of experienced difficulty by study.

Study	Social Class	Difficulty-as-Importance Condition			Difficulty-as-Impossibility Condition		
		% Agree	% Neutral	% Disagree	% Agree	% Neutral	% Disagree
1	High	68	11	21	3	8	89
2	Low	86	10	4	37	24	39
3	High	70	18	12	5	7	88
4	High	62	18	21	5	6	90

*Social Class High, Samples from Private or Flagship Public University; Social Class Low, Sample from 66% free/reduced price lunch school. % Agree, Neutral, and Disagree reflects mean response to four items on a 6-point scale (1, *strongly agree*; 2, *agree*; 3, *slightly agree*; 4, *slightly disagree;* 5, *disagree*; 6, *strongly disagree*). % Agree, mean score less than or equal to 3.0. % Neutral, means score greater than 3.0 (slightly agree) but less than 4.0 (slightly disagree) % Disagree, mean score greater than or equal to 4.0.*

### Study 2

#### Sample

Participants were sixth graders in eight language arts classes (*N* = 175) from a Detroit-area middle school serving low-income families (66% of student body receives free/reduced price lunch).

#### Procedure

Our agreement with the school was to come to class only once. Each student received a booklet entitled “Middle School and Beyond” and worked at his or her own pace on the materials during the approximately 30-min class period. We used the teacher provided classroom lists to randomize students into three groups (control, difficulty-as-importance, difficulty-as-impossibility). We discarded empty booklets from students who were not in class and booklets of students who skipped elements (*n* = 163 for analyses, difficulty-as-importance *n* = 51, difficulty-as-impossibility *n* = 59, control *n* = 53).

Booklets looked alike from the outside and differed only in one way. Inside of the cover were four “difficulty-as-importance” or four “difficulty-as-impossibility” statements, which were worded in a more didactic manner and simplified for a lower reading level in order to better suit the middle school setting (for exact wording, see Appendix located in Supplemental Materials). In the no interpretation of difficulty control condition, the inside of the cover was blank (default interpretation). The dependent variable was the standardized writing test that students would be taking that year (the Michigan Educational Assessment Program, MEAP). Students were asked to write an essay on the topic “Making a Difference” (topic, writing prompts, and time limit all followed the sample MEAP test we obtained). After writing the essay, final questions were: “*How hard did you try on your essay”* (1 = *I did not try at all*, 5 = *I tried very hard, M* = 3.78, *SD* = 1.04), age, gender, and expected Language Arts grade.

Mean writing quality (α = 0.96, *M* = 2.43, *SD* = 0.79) was obtained by averaging scores on the five MEAP writing criteria (*addresses the question topic, presents a thorough explanation, provides appropriate detail (no irrelevant detail), tells a coherent story with logical progression and flow, shows advanced writing ability*). Two independent raters (blind to condition) coded each criterion with the MEAP rubric (1 = *low / not proficient*, 4 = *advanced).* One rater coded all essays, the other double-coded 20% selected at random (*n* = 35), yielding high inter-rater reliability (*r* = 0.87). Raters also calculated each essay’s word (*M* = 94.39, *SD* = 53.79) and sentence (*M* = 6.65, *SD* = 4.48) counts.

Preliminary analyses showed that girls (*M* = 2.76, *SD* = 0.75) wrote better essays than boys (*M* = 2.03, *SD* = 0.65; *b* = 0.73, *p* < 0.001, 95% CI [0.51, 0.95]). Students who expected a higher grade in their class wrote better essays, *b* = 0.26, *p* < 0.001, 95% CI [0.14, 0.38]. Students who said they tried harder on the essay wrote better essays, *b* = 0.32, *p* < 0.001, 95% CI [0.21, 0.44]. Mechanics also mattered: students who used more words, *b* = 0.49, *p* < 0.001, 95% CI [0.39, 0.59] and more sentences, *b* = 0.46, *p* < 0.001, 95% CI [0.36, 0.56] wrote better essays. Hence these variables were included as controls in our regression analyses.

#### Results

In Study 2, the inclusion of a subjective measure of perceived effort allowed us to examine the effect of condition on students’ metacognitive experience of effort (experience of having tried) during the writing task. With regard to Contrast 1, we found no difference in subjective experience of effort when the contrast was between students in the difficulty-as-importance (*M* = 3.61, *SE* = 0.12) condition and other students (students in the difficulty-as-impossibility and control conditions combined), *b* = -0.21, *p* = 0.162, 95% CI [-0.51, 0.09]. With regard to Contrast 2, we found a significant difference in subjective experience of effort when the contrast was between students in the difficulty-as-impossibility condition (*M* = 4.07, *SE* = 0.12) and students in the control condition, *M* = 3.57, *SE* = 0.12; *b* = -0.50, *p* = 0.004, 95% CI [-0.83, -0.17]. These results suggest that students guided to a difficulty-as-impossibility mindset perceived their effort on the task as particularly strenuous, as might be expected if the prime sensitized them to the possibility that difficulty implied low odds of success.

**FIGURE 2:**
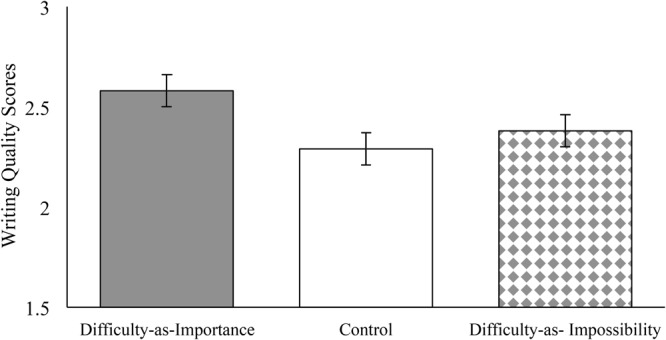
Study 2: Students’ Writing Quality scores by Condition (Difficulty-as-Importance, Control, Difficulty-as-Impossibility). Writing quality (1, *low/not proficient*; 2, *partially proficient*; 3, *proficient*; 4, *advanced*). Estimated marginal means include adjustments for covariates. Error bars represent standard errors.

If we look at performance, however, the feeling of effort induced by the difficulty-as-impossibility condition did not translate into higher quality essay writing. In the absence of the relevant control variables, essay performance does not significantly differ by condition (difficulty-as-importance vs. difficulty-as-impossibility and control combined *b* = 0.13, *p* = 0.353, 95% CI [-0.14, 0.39]; difficulty-as-impossibility vs. control, *b* = 0.05, *p* = 0.792, 95% CI [-0.25, 0.34]). However, once the relevant controls—the significant individual differences on essay performance—are included as covariates, a significant effect of interpretation-of-difficulty condition emerges. **Figure 2** presents average writing quality scores across the three conditions including the control variables detailed in the preliminary analysis section. With regard to Contrast 1, the quality of essays written by students in the difficulty-as-importance condition (*M* = 2.58, *SE* = 0.08) was better than the quality of essays written by students in the other conditions (control and the difficulty-as-impossibility) combined, *b* = 0.24, *p* = 0.013, 95% CI [0.05, 0.44], *d* = 0.31. With regard to Contrast 2, the quality of essays written by students in the control condition (*M* = 2.29, *SE* = 0.08) did not differ from the quality of essays written by students in the difficulty-as-impossibility condition (*M* = 2.38, *SE* = 0.08; *b* = -0.09, *p* = 0.425, 95% CI [-0.31, 0.13]).

As shown in **Table 2**, most participants in the difficulty-as-importance condition agreed that difficulty means importance (*M* = 4.76 *SD* = 0.86) and most participants in the difficulty-as-impossibility condition did not disagree that difficulty means impossibility (*M* = 3.40, *SD* = 1.11). If endorsement mattered, then rejecting difficulty-as-impossibility statements and accepting difficulty-as-importance statements should have parallel effects in the experimental groups (the control group was not shown interpretation of difficulty statements so could not be included in these analyses). To test this possibility, we entered mean endorsement scores into our regression equation. We coded our ‘difficulty-as-importance’ condition as +1 and our ‘difficulty-as-impossibility’ condition as -1 as we did in Study 1 and also entered mean endorsement of primed mindset into our regression equations for experience of effort and for essay quality. First we examined the effect of endorsing the primed difficulty mindset on subjective experience of effort finding no main effect of primed difficulty mindset endorsement, *b* = 0.06, *p* = 0.478, 95% CI [-0.11, 0.24] and no condition by endorsement interaction, *b* = 0.11, *p* = 0.258, 95% CI [-0.08, 0.29]. Then we examined the effect endorsing the primed difficulty mindset on essay quality, finding a significant effect of primed difficulty mindset endorsement, *b* = 0.13, *p* = 0.019, 95% CI [0.02, 0.24], and no interaction effect, *b* = 0.01, *p* = 0.916, 95% CI [-0.11, 0.12]. Essay quality was enhanced if students accepted the difficulty-as-importance interpretation they were guided to consider (almost all did) or if they rejected the difficulty-as-impossibility interpretation of difficulty they were guided to consider (only about 4 in 10 did). Notably, students in the difficulty-as-impossibility condition appeared to be particularly sensitive to their feeling of difficulty. These students may have ceased effort prematurely upon feeling that they had tried quite hard, as this feeling did not translate to more successful essays in the difficulty-as-impossibility group. Having no proffered interpretation of experienced difficulty (control group) was no worse than being guided to consider difficulty-as-impossibility.

### Study 3

#### Sample

University of Michigan undergraduates (*N* = 110) came to a computer lab running Qualtrics software to participate in a study for course credit.

#### Procedure

Participants completed a pattern-matching task for a different study and then were randomly assigned to condition (difficulty-as-importance *n* = 50, difficulty-as-impossibility *n* = 60) using the same protocol as Study 1. The dependent measure and demographics (race, gender, and age) followed. Then participants were thanked, debriefed and dismissed.

The dependent measure was the open-ended possible self and strategy measure (e.g., Oyserman and Saltz, 1993; Oyserman et al., 2006). Specifically, participants were asked to imagine the person they expected to become next year, to describe that person (space was provided for 3 open-ended descriptions) and to mark with a check, each description that they were currently working on. Then they were shown each of the checked descriptions and asked what they were doing now to become like that person in the coming year (open-ended). This two-step procedure was repeated, but this time, participants were asked to imagine the person they wanted to avoid becoming next year. The Appendix (located in Supplemental Materials) provides the full instructions. Two coders, blind to condition, content-coded responses into school and academics, personality, social relationship, health or physical traits, and material life style categories, using the coding dictionary^4^. The full coding dictionary is also available in our Supplemental Materials. We computed percentage scores by adding the total number of responses a student wrote in each category, dividing by the total number of responses the student wrote overall and multiplying by 100. For example, if a student wrote 4 possible selves and strategies related to academics out of 10 selves and strategies total, their percentage score for the academic category would be 40%. The content of interest, school and academics, was the most common response, comprising 40.6% of responses on average. Example responses coded as school and academic are: “*I will get into a prestigious medical school*” by “*working hard to attain good grades;*” “*To have a job that challenges and interests me*” by “*exploring possible majors through a variety of classes*.” The other categories were less common—personality traits 23.4% of responses, social-relational 18.1%, material lifestyle 10.9%, health or physical traits 8.3%. Computed Kappa inter-rater reliability ranged from a low of 0.50 for the less common categories to 0.70 for the school and academic responses central to our analyses. A rule of thumb is that scores in the range of 0.61–0.80 represent substantial agreement, those in the range of 0.40–0.60 represent moderate agreement (see Landis and Koch, 1977). Disagreements were discussed to consensus.

As in Studies 1 and 2, our first step was to examine differences between demographic groups on our dependent measure. We found that female participants wrote a smaller percentage of academically focused possible identities and strategies than males (*Exp(b)* = 0.78, *p* = 0.032, 95% CI [0.61, 0.98]) so gender was included as a covariate in the logistic regression analyses, described next. Our second step was to examine our dependent variable for outliers. We identified 1 outlier data point with a Standardized Pearson’s residual greater than 3.0 on the possible identities and strategies outcome, so we test for an effect of condition both including and excluding this outlier.

#### Results

**FIGURE 3:**
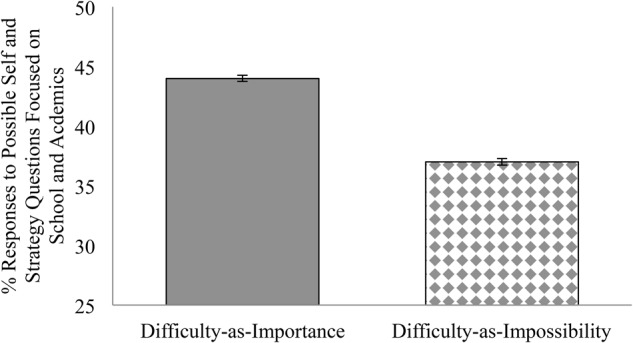
Study 3: % Responses to Possible Self and Strategy Questions Focused on School and Academics by Condition (Difficulty-as-Importance, Difficulty-as-Impossibility). Possible selves and strategies were content-coded and counted from an open-ended probe. Error bars represent standard errors.

Given that our dependent measure was a proportion of responses (academic-focused selves and strategies relative to total selves and strategies), we used SPSS’s GLM procedure’s logistic regression model to examine a binomial distribution, which allowed us to input both the number of academic-focused possible selves and strategies as well as the total number of possible selves and strategies reported by each participant. The prediction that difficulty-as-importance condition increases generation of academically focused possible identities and strategies relative to difficulty-as-impossibility condition was supported. Students in the difficulty-as-importance condition (*M* = 44%, *SE* = 2.1) generated more academically focused possible identities and linked strategies than students in the difficulty-as-impossibility condition (*M* = 37%, *SE* = 1.9), *Exp(b)* = 0.721, *p* = 0.006, 95% CI [0.57, 0.91]. This effect was robust, remaining when the effect of condition on academic possible identities and strategies was assessed without controlling for the significant individual difference of gender and retaining the outlier described in the preliminary analyses, *Exp(b)* = 0.748, *p* = 0.014, 95% CI [0.59, 0.94] as depicted in **Figure 3**. As displayed in **Table 2**, most participants in the difficulty-as-importance condition agreed that difficulty means importance (*M* = 4.04 *SD* = 0.72) and most participants in the difficulty-as impossibility condition disagreed that difficulty means impossibility (*M* = 2.00 *SD* = 0.86). If endorsement mattered, we should have found that rejecting difficulty-as-impossibility and accepting difficulty-as-importance had parallel effects. To test this possibility, we entered mean endorsement scores into our regression equation, finding neither a main (*Exp(b)* = 0.896, *p* = 0.146, 95% CI [0.77, 1.04]) nor an interaction effect of endorsement (*Exp(b)* = 1.110, *p* = 0.512, 95% CI [0.81, 1.52]). The implication is that when priming tasks are subtle, people are sensitive to the implications of the interpretation-of-difficulty mindset that is accessible and use it in considering what is possible for them even if they do not explicitly agree with this mindset. Two questions remain, first, how stable are these results, and second, do participants not guided to a particular interpretation-of-difficulty mindset more resemble difficulty-as-importance or difficulty-as-impossibility participants. To address these questions, we pre-registered a replication^5^ in which a control group was added.

### Study 4

#### Sample

University of Southern California undergraduates (*N* = 189) participated for course credit, coming to the lab and working on a computer using Qualtrics software.

#### Procedure

Participants completed a pattern-matching task for a different study and then were randomly assigned to condition (difficulty-as-importance *n* = 63, difficulty-as-impossibility *n =* 63, and no prime control *n* = 63). Participants then completed the same open-ended possible self and strategy measure used in Study 3 (e.g., Oyserman and Saltz, 1993; Oyserman et al., 2006). Two coders, blind to condition, content-coded 30 of the responses into school and academics, personality, social relationships, health or physical traits, material life style, and negative or non-normative categories, using the coding dictionary included in the Appendix (located in Supplemental Materials). Computed Kappa inter-rater reliability was high, ranging from 0.71 to 1, and disagreements were discussed to consensus. A second round of 10 responses were content coded by both coders, with even higher inter-rater reliability (ranging from 0.79 to 1), and again any disagreements were discussed to consensus. Therefore, one coder, blind to condition, content-coded the remaining responses. The content of interest, school and academics, was the most common response, comprising 35.4% of responses on average, while the other categories were less common – personality traits 22.1% of responses, social-relational 28.9%, material lifestyle 6.6%, health or physical traits 6.0%. Participants then completed demographics (race, gender, and age), were thanked, debriefed and dismissed.

Our first step was to examine differences between demographic groups on our dependent measure. We found that male participants wrote a smaller percentage of academically focused possible identities and strategies than females (*Exp(b)* = 0.79, *p* = 0.018, 95% CI [0.64, 0.96]). Note that this is reverse of the Study 3 pattern. Our second step was to examine our dependent variable for outliers. We identified 4 outlier data points, each with a Cook’s D greater than.05, which corresponded to a Standardized Pearson’s residual greater than 3.0. However, our as predicted pre-registration failed to document either the step of looking for demographic covariates or the step of examining the open-ended data for outliers. Hence, we ran our analyses twice, once failing to account for gender differences in the dependent variable and failing to exclude outliers and once with the gender covariate and without outliers.

We note that both strategies can be considered flawed: failing to include as covariates variables that influence the dependent variable and keeping outliers is considered inappropriate as outliers may distort results. At the same time including covariates and removing outliers when not documented in a preregistration is considered inappropriate as researchers may seek covariates and outliers as a way of changing results.

#### Results: Pre-registered Analyses Including Outliers and Without Gender as a Covariate

We ran a logistic regression model including the two contrast codes outlined in our analysis plan in the model. Both contrast codes were significant. Contrast 1 reflected an unexpected pattern, in which students in the ‘difficulty-as-importance’ condition (*M* = 33%, *SE* = 1.8) wrote fewer academically focused possible identities and strategies than students in the other two conditions combined, *Exp(b)* = 1.34, *p* = 0.009, 95% CI [1.08, 1.67]. Contrast 2 helps explain this pattern, in that the control group wrote a particularly large number of academic possible selves in this sample. Contrast 2 indicated a significant difference between the control and difficulty-as-impossibility conditions (*Exp(b)* = 0.770, *p* = 0.019, 95% CI [0.62, 0.96]). Participants in the ‘difficulty-as-impossibility’ condition wrote significantly fewer academically focused possible identities and strategies (*M* = 34%, *SE* = 1.8) than those in the control condition (*M* = 40%, *SE* = 1.9).

As in Studies 1 and 3 and displayed in **Table 2**, most participants in the difficulty-as-importance condition agreed that difficulty means importance (*M* = 4.04 *SD* = 0.99; without outliers *M* = 4.04 *SD* = 0.99) and most participants in the difficulty-as impossibility condition disagreed that difficulty means impossibility (*M* = 1.97 *SD* = 0.94; without outliers *M* = 1.91 *SD* = 0.89). To examine the potential effects of endorsement of either mindset, we entered mean endorsement scores into our regression equation, which was limited to the two interpretation-of-difficulty conditions. This analysis revealed a significant interaction effect of endorsement with condition (*Exp(b)* = 1.40, *p* = 0.004, 95% CI [1.11, 1.77]). To examine this interaction, we conducted additional regression analyses to determine how the effect of endorsement varied across condition and found that a significant, negative relationship emerged between endorsement and academic possible identities and strategies in the difficulty-as-impossibility condition (*Exp(b)* = 0.71, *p* < 0.001, 95% CI [0.60, 0.84]), while no relationship emerged in the difficulty-as-importance condition (*Exp(b)* = 1.00, *p* = 0.951, 95% CI [0.85, 1.17]).

#### Results: Follow Up Analyses Without Identified Outliers and Including Gender Covariate (to Parallel Study 3)

**FIGURE 4:**
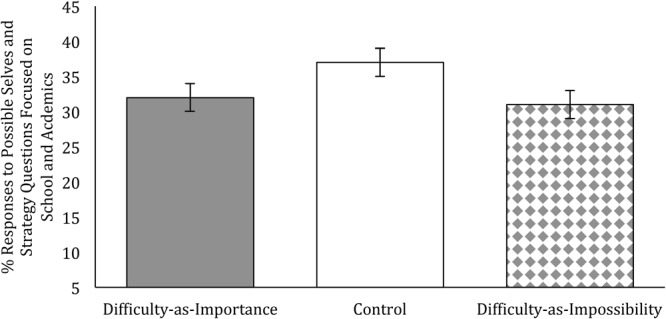
Study 4: % Responses to Possible Self and Strategy Questions Focused on School and Academics by Condition (Difficulty-as-Importance, Difficulty-as-Impossibility). Possible selves and strategies were content-coded and counted from an open-ended probe. Estimated marginal means include adjustments for covariates. Error bars represent standard errors.

We ran an additional set of analyses that included gender as a covariate and excluded four outlier data points. Though we failed to pre-register this plan; it can be considered the more appropriate plan given preliminary analyses showing a significant gender difference and four outliers on our open-ended dependent measure (Standardized Pearson’s residual greater than 3.0, Cook’s *D* greater than 0.05) and that this analyses parallels the analyses in Study 3. We again examined the effect of accessible mindset, running a logistic regression that included the two contrast codes described above along with gender. This model revealed a significant Contrast 2, which tested the comparison between the control and difficulty-as-impossibility conditions (*Exp(b)* = 0.779, *p* = 0.030, 95% CI [0.62, 0.97]). As depicted in **Figure 4**, participants in the ‘difficulty-as-impossibility’ condition wrote significantly fewer academically focused possible identities and strategies (*M* = 31%, *SE* = 1.8) than those in the control condition (*M* = 37%, *SE* = 1.9). Contrast 1 was not significant. Contrast 1 showed a non-significant unexpected pattern in which students in the ‘difficulty-as-importance’ condition (*M* = 32%, *SE* = 1.8) wrote fewer academically focused possible identities and strategies than students in the other two conditions combined, *Exp(b)* = 1.24, *p* = 0.056, 95% CI [0.99, 1.55], clearly driven by the high percentage of academic possible selves in the control group. We also conducted a simplified regression analysis that excluded the control condition in order to mirror the analysis in Study 3, which also included a gender covariate and excluded outliers. In these analyses we found no significant effect of condition, *Exp(b)* = 0.973, *p* = 0.810, 95% CI [0.78, 1.22].

We again examined the effect of endorsement by entering the centered mean endorsement variable into our regression equation, and found neither a main (*Exp(b)* = 0.91, *p* = 0.105, 95% CI [0.80, 1.02]) nor an interaction effect of endorsement (*Exp(b)* = 1.16, *p* = 0.251, 95% CI [0.90, 1.48]). This endorsement pattern was similar to that of Studies 1 and 3. Considering Studies 3 and 4 together, it appears that the effect of endorsement of difficulty as impossibility on number of generated possible selves and strategies is not stable.

## Discussion

In two experiments we showed downstream consequences for academic engagement and performance of accessible interpretation of experienced difficulty (as implying value and importance versus low odds of success and impossibility) separate from endorsement of that interpretation. In one study, college students were better and more efficient at finding correct solutions to a difficult academic task (Study 1). In a second study, middle school students in a low social class context scored better on a standardized writing task when proffered an interpretation of experienced difficulty as importance than when proffered no interpretation of experienced difficulty at all (control group). Being given no interpretation was like being proffered a difficulty-as-impossibility interpretation of experienced difficulty. We make that inference because these two groups scored no differently, suggesting that students may assume that difficulty implies impossibility when not offered an alternative. Indeed, in this low social class context, most students in the difficulty-as-impossibility condition did not explicitly reject that message, unlike students in the high social class contexts. While accepting a difficulty-as-importance message and rejecting a difficulty-as-impossibility message predicted better performance, endorsement did not moderate the main effect of proffered interpretation of experienced difficulty.

In two other experiments we showed inconsistent effects for salient content of the future self. In one study, college students proffered an interpretation of experienced difficulty-as importance-rather than as impossibility had more school-focused possible identities and strategies to attain them (Study 3). This main effect of proffered interpretation was not moderated by how much students explicitly endorsed the message in the mindset they were proffered. Students in the difficulty-as-impossibility condition were less school-focused in their identities even though most of them rejected the difficulty-as-impossibility message. The pattern of rejecting the difficulty-as-impossibility message and accepting the difficulty-as-importance message was consistent in our registered replication. However, in this registered replication at an even more elite college campus, we did not find a consistent effect of proffered interpretation of experienced difficulty; a proffered interpretation of difficulty as impossibility did undermine focus on academic possible identities compared to control group but a proffered interpretation of difficulty as importance did not increase focus on academic possible identities. Hence future work is needed to understand whether cuing interpretation of experienced difficulty as importance is helpful among elite college students.

### Syntheses and Contribution to Existing Literatures

Taken together, our results add to the current literature on motivating performance in several ways. First, our results add to the literature on identity-based motivation (e.g., Oyserman et al., 2017) by showing effects of accessible interpretation of experienced difficulty on academic performance on standardized tests and identities. In doing so we address a gap in prior research, which showed effects on identity and performance as a result of interventions intended to change social norms about interpretation of experienced difficulty (Oyserman et al., 2002, 2006; Smith and Oyserman, 2015). Prior studies (Aelenei et al., 2016; Elmore et al., 2016) found that students needed to endorse productive interpretations—that difficulty implies importance or that difficulty does not imply impossibility—when accessibility was induced in ways that led them to question why an interpretation was on their mind. Our results expand on this work by showing a main effect of accessible interpretation separate from endorsement if guided interpretation is subtle. In these subtle influence conditions, the effects of guided interpretation of experienced difficulty were not moderated by endorsement. This is particularly interesting because students generally disavow the idea that their experienced difficulty might mean that a task might be impossible for them. These results point to accessibility as the mechanism through which these effects occur—because students did not actively question why a particular interpretation came to mind, they instead used the most easily accessible interpretation to respond to difficulty.

These studies also replicate and expand on prior research showing higher endorsement of difficulty-as-impossibility statements among low-income participants (Fisher and Oyserman, 2017). We show that in a low-income context, students benefited from being guided to consider a difficulty-as-importance mindset, whereas being guided to consider a difficulty-as-impossibility mindset was indistinguishable from being proffered no interpretation at all. This is true even though most low-income students accepted or were neutral about the idea that difficulty implies impossibility of success. These findings imply that social class effects on identity-based motivation are likely to be nuanced (see also Fisher et al., 2017).

In addition, our results add to literature on the effects of metacognitive experience (Koriat, 2008; Schwarz, 2015). As applied to academic performance, metacognitive experiences of ease and difficulty contribute to feelings of knowing, learning, and remembering (Bjork et al., 2013). Students may misread their metacognitive experience of ease or difficulty with study materials as having implications for whether they already know the material; for example, experienced ease may be misinterpreted as knowing, so students stop studying (Koriat et al., 2009). Upon encountering difficult material, students who think that they are choosing to invest will study harder than those who think that they are forced to work harder because the material is more difficult (Koriat et al., 2014, see also Miele and Molden, 2010; Autin and Croizet, 2012). Our results show that when a difficulty-as-impossibility mindset was on the mind, students perceived their effort as particularly high compared to those in the difficulty-as-importance and control conditions. Perhaps a difficulty-as-impossibility mindset sensitized students to any effort as a sign that the work was particularly difficult. But this did not prompt continued effort and investment and did not translate into improved performance. Our results show that guided interpretation of experienced difficulty matters for how students make sense of their metacognitive experiences—students who are guided to consider difficulty-as-importance perform better on difficult academic tasks than students who are guided to consider difficulty-as-impossibility.

Next, our results add to literature examining motivation through a variety of distinct processes including self-efficacy, expectancy-value, growth and grit theories that highlight the motivational consequences of believing that one has or could have the skills needed to do well in school, believing that one is good at school, and valuing school (Feather, 1982; Eccles et al., 1983; Wigfield and Eccles, 1992; Bandura and Locke, 2003; Harackiewicz and Hulleman, 2010; Duckworth and Gross, 2014; Paunesku et al., 2015). Though each is distinct, these variables are also associated; for example, intervening to increase the value of education also increases school engagement (Harackiewicz et al., 2012, see also Duckworth and Seligman, 2005; Fan, 2011; Fisher and Oyserman, 2017). Prior research suggests that difficulty-as-importance scores provide a distinct addition to other constructs in understanding how motivation works, separate from growth mindset, efficacy, and grit (Fisher and Oyserman, 2017). Our results suggest that guiding students to a difficulty-as-importance interpretation of their experienced difficulty provides a route to improved academic engagement and performance.

Lastly, our results add to literature on the interplay between identity-based motivation and the effects of stereotype threat (for a review, Oyserman and Lewis, 2017). Stereotype threat arises when an accessible stereotype provides an explanation for experienced difficulty, resulting in impaired performance among stigmatized individuals on standardized academic tasks such as the Graduate Record Exam (Aronson and Dee, 2012; Jamieson et al., 2012). Our results provide a general explanation for these effects, which is that experienced difficulty impairs performance in part by evoking a difficulty-as-impossibility mindset, even if one explicitly rejects this interpretation.

### Limitations and Implications for Future Research

Any set of studies has limitations and our studies are no exception. In this section we consider three main limitations of the current studies that have implications for future research: measurement, generalizability, and contextual sensitivity. Our experiments focus on a subtle manipulation and measure immediate effects, thus the duration of these effects cannot be determined from the current studies. Hence, it would not be appropriate to assume that an immediate effect on performance or future identity content continues over time without specific evidence of longevity of effects. In addition, while we documented an effect of accessible interpretation-of-experienced-difficulty mindset on which identities are salient in the moment can sometimes be detected with open-ended measures as well as with close-ended measures used in prior research (Smith and Oyserman, 2015; Aelenei et al., 2016), open-ended measures can be challenging to code and our results were not stable. Difficulty-as-impossibility definitely undermines identities but difficulty-as-importance may not always bolster them and effects may depend on chronic or immediate contextual cues. Finally, we focused on identity and performance and did not also include related motivational constructs such as growth mindset or grit as Fisher and Oyserman (2017) did. Although theoretically distinct, including these constructs in future work may uncover useful individual differences or moderation patterns that were unexplored in the current set of studies. Hence, future work considering how interpretation-of-experienced-difficulty mindsets might interact with other motivational mindsets could yield useful theoretical and practical insights.

Our experiments documented main effects of accessible interpretation of experienced difficulty in the U.S. in samples that differed by developmental period, social class, and race-ethnicity. We know that some elements of identity-based motivation (e.g., effects of accessible academic possible identities on action readiness) are stable across cultures and contexts (Zhu et al., 2014; Bi and Oyserman, 2015). While important, we cannot yet tell whether our results would replicate in different cultural settings. We are beginning to test this question using a large sample of Chinese middle school students, replicating the effect of accessible interpretation of experienced difficulty but also showing that students are equally influenced whether the interpretation is their own, their teachers’, or their parents’ (Bi and Oyserman, 2016, Unpublished). Our current studies cannot address the question of whether accessible interpretations are of more or less or equal consequence across cultures. Hence future research is needed to test the possibility that a difficulty-as-importance interpretation might be more chronically accessible in some settings and difficulty-as-impossibility might be more chronically accessible in other settings that may differ in which interpretations of experienced difficulty feel culturally fluent (Oyserman, 2017; Oyserman and Yan, 2018). This might depend on resources; prior findings and our own results imply that low position in a social class hierarchy makes difficulty-as-impossibility interpretations more chronically accessible, though no less amenable to intervention. However, our samples differed in age as well as social class, and age might also matter—for example, younger students might be differentially sensitive to influence attempts or differentially likely to have a chronic interpretation of experienced difficulty on the mind. Personal resources might also matter—our results show effects of guided interpretation of experienced difficulty on subsequent performance. Given their experimental nature, our results cannot speak to the possibility that guided interpretation of experienced difficulty-as-importance might be particularly beneficial for students with worse track records of success or more uncertainty about their future success.

What we did show with regard to contextual sensitivity is that students are sensitive to contextual cues, taking on interpretations of experienced difficulty introduced in a set of four statements that they were simply asked to read and rate. Theory suggests that an accessible difficulty-as-impossibility interpretation is productive in circumstances in which persisting is unlikely to help and alternatives are available to be discovered. Other research has documented that it is sometimes better to shift attention to something else or risk overinvesting in a failing enterprise (Arkes and Ayton, 1999; Walton, 2002) or perseverating on unattainable goals (e.g., Wrosch et al., 2003a,b, 2007; Miller and Wrosch, 2007). While we were able to show that we could shift interpretive lens, our academically focused dependent variables did not test the possibility that using a difficulty-as-impossibility lay theory can be helpful when it is time to quit a goal, this too is a potentially productive path for future research.

That a difficulty-as-impossibility mindset can sometime be helpful can be seen in the following example. Consider a student who starts college with a planned major, but attains disappointing grades in required classes for this major. Initially, a difficulty-as-importance mindset might be the better choice. Interpreting experienced difficulty as importance can ratchet up effort, increasing chances of success. Yet at some point, switching to a difficulty-as-impossibility mindset might be more productive. After all, interpreting experienced difficulty in terms of low likelihood of success in that particular planned major might facilitate success in a larger goal of graduating with high enough grades to be employable. An accessible difficulty-as-impossibility mindset might be needed to free up attention to seek out an alternative path. Just as shifting effort away too soon is costly in terms of missing opportunities that would have arisen with persistent engagement; shifting too late or not at all might be costly as well, for example if the resultant grade point average makes the student uncompetitive for graduate school or employment.

Our results suggest that brief intervention can shift which interpretation of experienced difficulty is accessible in the moment with consequences for how students think about their futures, the number of strategies that come to mind to take action to attain these future, and their actual performance. Our results focus on immediate consequences of interpreting difficulty as importance – we do not assume that a brief priming task is sufficient for effects to last over significant periods of time. Our results suggest that efforts to remind students that difficulty can signal the important value of their work may keep more students on the path to academic success. We are testing this prediction in field-based research including activities meant to create an accessible difficulty-as-importance mindset (Oyserman and Sorensen, 2014; Horowitz et al., 2018).

## Ethics Statement

This Study (Study 1-3) was approved by the University of Michigan IRB, studies with college students had waiver of documentation of informed consent since data were collected as part of subject pool and students were told that participation was voluntary, that they could skip any item they did not wish to answer, and could stop at any time and data were non-sensitive and anonymous. Study 4 was approved by the University of Southern California IRB.

## Author Contributions

Studies 1, 2, and 3 were run as part of doctoral (KE and GS) and postdoctoral (SN) training and dissertation (GS). The authors (DO, KE, GS, and SN) collaborated: developing the study designs, initial analyses, and initial write up. Study 4 was pre-registered and run by OF. DO, KE, and OF together edited the final versions of the paper. In Study 4 KE coded open-ended data (blind to condition) and DO double coded a portion for reliability (blind to condition). KE conducted data analyses with input from DO and reviewer suggestions. All authors have read and signed off on this final version of the paper and are accountable for results. KE conducted final analyses and Figure development, with DO supervision, and was deeply involved with DO on all revisions of this paper. SN supervised Studies 1 and 3 (data collection for, open-ended coding of data in Study 3, initial analyses). GS collected data and supervised coding of open-ended data in Study 2, performed double code for reliability, and performed initial analyses.

## Conflict of Interest Statement

The authors declare that the research was conducted in the absence of any commercial or financial relationships that could be construed as a potential conflict of interest.

## Footnotes

1 Results show that students are sensitive to whether they seem to be interpreting their experienced difficulty as importance (impossibility) relatively more or less than others. Interpreting difficulty as importance more than others and interpreting difficulty as impossibility less than others is motivating.

2 This method has been recently used to test the effect of accessible theory about whether effort is depleting (ego depleting) or energizing (Job et al., 2010) and the effect of accessible lay theory about what failure means (Haimovitz and Dweck, 2016). In that work, researchers randomized participants to two groups. One group read and rated endorsement of a set of items describing one theory (e.g., willpower as a fixed resource). The other group read and rated endorsement of a set of items describing the other theory (e.g., willpower as a renewable resource). Then both groups completed a task (the dependent variable). Results demonstrated that the lay theory brought to mind in the set of items in the prior task influenced subsequent performance.

3 https://www.openicpsr.org/openicpsr/project/102262/version/V1/view/

4 https://dornsife.usc.edu/assets/sites/782/docspossible_selves_measure.doc

5 http://aspredicted.org/blind.php?x=z5my4q
