# Supplementary material for: Guiding People to Interpret Their Experienced Difficulty as Importance Highlights Their Academic Possibilities and Improves Their Academic Performance
Source: Front Psychol. 2018 May 25;9:781. doi: 10.3389/fpsyg.2018.00781 (PMC5983065; doi:10.3389/fpsyg.2018.00781)
Supplement: Supplementary file 1 [file Data_Sheet_1.docx]

**APPENDIX**

We list below the specific interpretation of experienced difficulty mindset primes used in each Study and the open-ended possible identity measure used in Studies 3 and 4.

**INTERPRETATIONS OF EXPERIENCED DIFFICULTY**

Please indicate how much you agree or disagree with each of the following statements by selecting the response that corresponds most closely to your opinion.

1= strongly disagree, 2= disagree, 3= slightly disagree, 4=slightly agree, 5= agree, 6= strongly agree.

**Difficulty-as-importance**

**Studies 1, 3, and 4**

1. Some school tasks feel easy and some feel difficult. My gut tells me that if it feels difficult, it is important for me.
2. I know in my gut that if a school task feels difficult it is really important for me.
3. I know that goals at school that feel difficult are the important ones for me.
4. A feeling of difficulty means that it’s probably an important school task for me.

**Study 2**

1. When you find yourself working really hard on a school task, it’s okay. That feeling just means it’s important to you.
2. You can use your feelings about working on a school task to tell you how important it is for you. If you keep working even when it feels hard, it’s probably important to you.
3. As a student, you know that difficult goals are the important ones. On those tasks, difficulty means that you should work harder.
4. Sometimes you have to work really hard in order to be successful at a school task, and there’s nothing wrong with that.

**Difficulty-as-Impossibility**

**Studies 1, 3, and 4**

1. Some school tasks feel easy and some feel difficult. My gut tells me that if it feels difficult, it is impossible for me.
2. I know in my gut that if a school task feels difficult it is really not possible for me.
3. I know that goals at school that feel difficult are the impossible ones for me.
4. A feeling of difficulty means that it’s probably an impossible school task for me.

**Study 2**

1. When you’re stuck on a school task, it is a sign that your effort is probably better spent elsewhere.
2. Sometimes, working on a school task feels very difficult – impossible really. That’s okay, because finding out that you are not likely to be successful can be helpful for moving on to other tasks.
3. Students shouldn’t waste time on tasks that just aren’t meant for them.  If a task feels too hard, then you should move on to something else so that you can succeed in something else.
4. As a student, you know that when working on a school task feels hard, that feeling means that it might just not be for you.

**POSSIBLE IDENTITIES AND STRATEGIES TO ATTAIN THEM**

**Studies 3 and 4**

Students read, “Each of us has some mental image of the person we are now as well as positive and negative mental images of the person we might become in the future. Take a minute to think about the coming years and the person you are expecting to become. Think about the next few years of your life and then write down three descriptions of the person you are expecting to become during these years. Your description could focus on things you will be doing, roles you will take on, traits you will have, or anything else.” Then they were asked to describe what they are doing to attain that self in the future.

Then students read, “Each of us has a mental image of the person we hope to avoid becoming or avoid being like. Take a minute to think about the coming years and the person you are concerned you may become. Think about the next few years of your life and then write down in the three descriptions of the person you are concerned you may become during these years. Your description could focus on things you will be doing, roles you will take on, traits you will have, or anything else.” Students were then asked describe what they are doing to avoid that self in the future.

# CODING INSTRUCTIONS FOR NEXT YEAR POSSIBLE SELVES

**STUDIES 3 & 4**

1. **CATEGORY LABELS**

There are six main categories of Next Year Expected Possible Selves:

1. **Achievement**- relates to school and school interactions with teachers, achievement-related activities
2. **Interpersonal Relationships**- involves family, friends, relationships, and social interactions except with teachers
3. **Personality Traits**- relates to personality characteristics, self-descriptions of traits
4. **Physical/Health-Related**- relates to physical health, weight, height
5. **Material/Lifestyles**- relates to material possessions and living situation, including moving
6. **Negative**- includes all negatively worded responses

There are six main categories of Next Year Feared Possible Selves.

1. **Achievement**- relates to school and school interactions with teachers, achievement-related activities
2. **Interpersonal Relationships**- involves family, friends, relationships, and social interactions except with teachers
3. **Personality Traits**- relates to personality characteristics, self-descriptions of traits
4. **Physical/Health-Related**- relates to physical health, weight, height
5. **Material/Lifestyles**- relates to material possessions and living situation, including moving
6. **Non-normative /Risky Behaviors**- includes negative and illegal behaviors such as smoking, drinking, involved in fights, gangs, etc.
7. **SUMMARY OF CODING**

Categories of expected and feared possible selves are identical except that the sixth category for expected selves includes ANY negative reference (since the vast majority of expectations are framed positively) and the sixth category for feared possible selves includes behaviors or expectations that are either delinquent or risky behaviors (such as teen pregnancy or smoking). Each category includes subcategories that are listed below. These may be useful for analyses though in our work to date sample size has been such that we have focused on main categories only.

1. **CODING AMBIGUOUS STATEMENS**
   1. CONSIDER AGE OF RESPONDENT -- When coding for possible selves, one must first consider the age of the respondent. The same response e.g. “getting my license” may be either a codable or noncodable response depending on they respondent’s age (that is NEXT YEAR is this possible?). For example, when an eighth grader expects to be a doctor that response is not coded. In a very few instances, age may also determine which category the possible self is placed. For example, when a twelve-year old respondent reports that next year he/she would like to avoid smoking, this feared possible self is categorized as non-normative. For an older person (16 and above), this same feared self would be coded in the health category.
   2. CONSIDER CONTEXT OF RESPONSE -- When a possible self is ambiguous because too little has been written, read through the strategy provided for that possible self to see if it provides clues for the content intended.
2. **EXAMPLES OF NEXT YEAR POSSIBLE SELVES BY CATEGORY**

**1. Achievement**

Expected selves:

- Job- working for extra money, finding summer job, working, help mom save for school, babysitting, having a job, part-time job
- Activities in school- cheer team, basketball team at school, playing instrument, school band, extra-curricular activities, playing sports, on a team, a better basketball player, getting a driver’s license
- School- doing good in school, trying to do good in school, smart, getting good grades, going to the next grade, keep my grades up, not tardy or absent from school, more helpful in classroom, honor roll, good conduct, going to better/new school
- Teachers- good relationship with teachers, getting along with teachers, respectful to teachers
- Activities Not in School- basketball in neighborhood, guitar, deer hunting, reading a lot of books, boxing, (Note: Generally, if there is any doubt about activities put in school activities except for things not offered in school)

Feared Selves:

- Job-losing my job, without work
- Activities in school- not on team, not making cheerleading
- School- known as bad kid by teachers, a loser, dropout, flunking out of my classes, having bad grades, dumb, having bad schoolwork, not paying attention, not falling behind in class, in trouble in school, being a little punk, fighting in school, suspended, excluded, skipping, in same grade
- Teachers- still getting trouble with teachers, back talking to teachers,
- Activities Not in School-I don’t want to be home all the time

**2. Interpersonal Relationships**

Expected selves:

- General- shy, silly, nice, respectful, better listener, funnier
- Family- obedient, getting along with parents/relatives, helping around house, better person towards mother, see relatives, doing things with family, closer to family, being a good/ better son/daughter
- Peers- having a steady boyfriend, getting along better with people, having lots of friends/same friends, making new friends, having lots of friends, hang with friends more, trying to be accepted at new school, being a better friend

Feared selves:

- General- as shy as I am, avoid being a recluse, mean person, rude, stuck up, mistrusted, stingy,
- Family- having anything happen to our family, not listening to parents, being smart with parents, mean to sibling/relative, getting into arguments with parent/relative, without someone to turn to
- Peers- enemies with other people, being a follower, being disliked by friends, not making friends, lying to people, boring, very talkative, breaking up with girl/boyfriend, bully, troublemaker, bad to my friends, without friends because of rumors, used just for my car-for rides

**3. Personality Traits**

Expected selves:

- Independence or Maturity- more mature, more responsible, more grown-up, helping her without complaining, A little more organized, Able to concentrate
- Attitude- more serious person, being more open-minded, positive thoughts, positive attitude, to be a good person

Feared selves:

- Independence or Maturity- lazy, irresponsible, not trusted
- Attitude- a bad attitude, silly, greedy, weak mentally, emotional mess, caring about nothing

**4. Physical**

Expected selves:

- General Body Descriptive-Hair looking different, as short as I am this year, taller, growing a few inches, Handsome, good-looking,
- Physical Health- older, 15 years old

Feared Selves:

- General Body Descriptive- Getting my haircut, wearing dark lipstick like a devil worshipper, Short, I want to grow,
- Physical Health- Sick a lot, so sick I can’t attend school, On medication again, Slower physically, weaker than I am, overweight

**5. Material/ Lifestyle**

Expected selves:

- Lifestyle-Still living at home with my mom, moving to Canada, living somewhere, going places I have never been,
- Material-own a car, living in better new house

Feared Selves:

- Lifestyle- Moving from this house
- Material- In the situation of money, not money confused

**6. Negative**

Possible selves responses that are worded negative or suggest an expected negative outcome should be placed in the negative category. The response could be categorized from any of the 6 possible selves categories. For example, students may respond “next year, I expect to still be involved in fights (negative-delinquent), or I expect to have few friends (negative-interpersonal relationships). However, these statements are sometimes worded in a negative form. For example, a student may respond “next year, I expect to be not fighting (negative-delinquent), or not to be getting picked on (negative-interpersonal relationships).

**6. Non-Normative**

Expected selves:

- Do not use for expected selves-Use Negative Category

Feared Selves:

- Non-normative- getting pregnant, cigarettes, being killed, hanging out with wrong people, troublemaker, having sex
- Delinquent- shooting people, with gang members, getting involved in drugs, In a gang, gang banger, alcohol use, a druggie, drug dealer, jail

**E. INSTRUCTIONS FOR CODING STRATEGIES**

When a strategy or strategies is/are given for each possible self, the strategy should be coded in the same category as the possible self. The categories are the same as the expected and feared possible selves categories: 1) Achievement, 2) Interpersonal relationships, 3) Personality Traits, 4) Physical/Health-Related, 5) Material/lifestyles, 6) Non-Normative, 7) Negative.

**Examples of Strategies:**

**1. Achievement:**

- Working hard on assignments
- Doing all my schoolwork
- Paying more attention

**2. Interpersonal Relationships:**

- Doing what others tell me to do
- Working with parents
- Asking for help

**3. Personality Traits:**

- Controlling my attitude/actions
- Trying new things
- Disciplining myself

**4. Physical:**

- Lifting weights
- Exercising
- Eating healthy foods

**5. Material/ Lifestyle:**

- Working to save my money
- Talking with parents about moving

**6. Non-normative:**

- Avoid being around negative/criminal people/activities
- Walk away from negative pressure situations
